# Identification of DNA methylation biomarkers in amniotic fluid for prenatal detection of congenital heart disease (CHD)

**DOI:** 10.1186/s13148-026-02090-4

**Published:** 2026-02-26

**Authors:** Weilun Zuo, Jianwei Rao, Yaqin Ma, Shiyu Sun, Xiaoqin He, Han Yang, Jiali Cao, Qichang Wu, Huiming Ye

**Affiliations:** 1 Department of Laboratory Medicine, Fujian Key Clinical Specialty of Laboratory Medicine, Women and Children’s Hospital, School of Medicine, Xiamen University, Xiamen, Fujian Province China; 2 Center of Prenatal Diagnosis, Women and Children’s Hospital, School of Medicine, Xiamen University, 10 Zhenhai Rd, Siming District, Xiamen City, Fujian Province China; 3 Department of Ultrasound, Women and Children’s Hospital, School of Medicine, Xiamen University, Xiamen, Fujian Province China

**Keywords:** DNA methylation, congenital heart disease, biomarkers, amniotic fluid, prenatal detection

## Abstract

**Background:**

Congenital heart disease (CHD) is the most common birth defect worldwide, affecting approximately 1% of live births. Improving the prenatal diagnosis of CHD remains an urgent clinical priority. In this study, we aimed to screen and validate amniotic fluid methylation biomarkers from clinical cases for the prenatal diagnosis of CHD.

**Methods:**

A total of 135 amniotic fluid samples (80 cases and 55 controls) collected during the second trimester were included and divided into three independent cohorts. Cohort I was used to screen for differentially methylated regions (DMRs) by whole-genome bisulfite sequencing (WGBS) during the discovery phase. The above candidate DMRs were detected using target bisulfite sequencing (TBS) in an independent cohort II. The final biomarker set was selected according to adjusted *P*-values, sequencing depth, group differences, and relevance to cardiac development and key metabolic pathways. The model was built using individual biomarker cut-off values. Its predictive performance and generalizability were evaluated in an independent cohort III.

**Results:**

WGBS and unsupervised clustering analysis revealed differential methylation patterns in amniotic fluid DNA between the CHD and normal groups. The 52 DMRs (adj *P* < 0.01) were identified by screening with the above factors. Then, the analysis of cohort II via TBS identified 25 differentially methylated sites. From these, a predictive model was constructed using the four hypermethylated sites with the greatest group differences, all located within a single intron of the *PCNT* gene. Validation in an independent cohort III identified 11 differentially methylated sites, with an 8-site overlap with cohort II. Notably, 7 of the overlapping sites were associated with *PCNT*, highlighting strong reproducibility. This model showed consistent and decent performance in the cohort III (specificity: 95.65%, sensitivity: 70.45%), the cohort II (specificity: 73.08%, sensitivity: 83.33%), as well as in simple congenital heart disease (SCHD) (specificity: 83.67%, sensitivity: 76.09%) and complex congenital heart disease (CCHD) sample sets (specificity: 83.67%, sensitivity: 75.00%).

**Conclusions:**

DNA methylation profiles in amniotic fluid differ significantly between fetuses with CHD and normal fetuses. The methylation of *PCNT* was suggested to be associated with the pathogenesis of CHD. Furthermore, the four-marker methylation panel identified in this study demonstrated decent efficacy in distinguishing fetuses with CHD, highlighting its promise as a new adjunctive diagnostic approach for prenatal CHD in high-risk pregnant women.

**Supplementary Information:**

The online version contains supplementary material available at 10.1186/s13148-026-02090-4.

## Background

Congenital heart disease (CHD) is generally defined as a birth defect that presents as a structural abnormality of the heart and/or great vessels. CHD is the most frequently occurring congenital disorder affecting approximately 1% of live births [1]. Although the introduction of large-scale intracardiac repair has significantly improved long-term survival rates for some patients since the 1950s, CHD remains the leading cause of mortality from birth defects [2]. A well-organized and generally accepted scheme classified CHD patients into highly complex, moderate severity and simple forms [3]. Complex CHD is associated with substantial mortality, whereas even surgically repaired simple CHD necessitates lifelong specialized care for survivors and carries risks of long-term complications [4].

Timely diagnosis of CHD is pivotal for early treatment and reduction of sequelae [5]. Therefore, prenatal diagnostic testing for congenital heart disease offers an effective means of prevention, which provides time for parental counselling, guiding the timing and optimal location of delivery, and postnatal stabilization before surgery [6]. The currently well-established method for the prenatal diagnosis of CHD is the second-trimester anomaly scan (SAS), which is based on fetal echocardiography and is limited by personnel experience, medical equipment and limitations of the technique itself [7, 8]. Thus, although the introduction of fetal echocardiography has further improved the outcome of CHD infants, many cardiac anomalies are still overlooked, resulting in detection rates ranging from approximately 6 to 35% [2, 7]. To improve the reliability of prenatal diagnostic testing for CHD, the development of a highly sensitive and specific detection method for the early diagnosis of CHD is urgently needed [7].

DNA methylation is a widely studied epigenetic modification, which is a covalent modification of DNA molecules that plays a broad role in the regulation of gene expression and genome stability [9]. This mechanism can reflect both genetic variation and environmental exposure, which are considered critical parts of the pathogenesis of CHD [10, 11]. In addition, DNA methylation is one of the longest-studied epigenetic mechanisms and is stable and relatively easy to measure [12, 13]. Given its multifaceted effects and relative ease of measurement, there is great potential for DNA methylation to be used as a biomarker. A thorough review [14] described the contribution of aberrant DNA methylation to CHD, and recent studies have also suggested the potential of DNA methylation as a prenatal marker for CHD [15].

As shown in Table 1, previous whole-genome epigenetic studies on CHD primarily utilized biological samples available at birth or postnatal, such as placental [16], umbilical cord blood [17], or peripheral blood from infants [18] and children [19]. While invaluable for elucidating etiology and identifying postnatal biomarkers, these sources are inherently limited for prenatal diagnosis due to their timing (post-delivery) or indirect relationship to the developing fetal heart. Maternal blood cell-free DNA [20], though promising for non-invasive screening, reflects a mixture of maternal and placental signals. As the direct environment for fetal growth, maternal amniotic fluid contains mainly fetal shed cells and urine [21]. Although limited by DNA abundance, it remains an ideal material for studying the methylation patterns of genes related to fetal heart development. However, it is increasingly feasible to detect amniotic fluid because of technological advances in methods for DNA isolation and detection of specific methylated DNA fragments in ever-smaller amounts of starting material [22].

Therefore, we hypothesized that amniotic fluid would harbor a distinct DNA methylation signature associated with fetal CHD. To our knowledge, no prior study has established a validated amniotic-fluid methylation panel for CHD. This study aimed to explore the associations of second trimester methylation biomarkers in amniotic fluid with fetal congenital heart disease. We first used whole-genome bisulfite sequencing (WGBS) to compare genome-wide DNA methylation patterns in fetuses with and without CHD. Two rounds of independent validation were then performed via target bisulfite sequencing (TBS), and four CpG sites were identified as a biomarker panel with preferable sensitivity and specificity, indicating robust performance in the diagnosis of CHD.

**Table 1:** Prior genome-wide methylation studies in CHD

Study	Sample type	CHD number	Sample collection stage	Differential methylation sites	Effect sizes	Sequencing platform
Radhakrishna et al. [16]	Placental tissue	8 isolated VSD	Postpartum	80 CpG sites	AUC = 1.0 (80 sites); others AUC ≥ 0.81	Illumina HumanMethylation450 Bead Chip
Bahado-Singh et al. [23]	Newborn dried blood spots	Various CHDs total *n* = 60 (HLHS, VSD, ASD, CoA, PS, TOF)	Newborn period	Multiple CpG sites (e.g., LASS3, GSTM1, etc.)	CoA detection: AUC = 0.974	Illumina HumanMethylation450 Bead Chip
Yuan et al. [17]	Umbilical cord blood DNA	Three pairs of monozygotic twins	At birth	SPESP1 and NOX5 promoters; DMRs in CERS1 and GDF1	Hypermethylation in CHD (*p* < 0.05)	WGBS
Radhakrishna et al. [18]	Newborn blood	24 (non-syndromic TOF)	24–79 h post-birth	TSPAN19, LHX9, MYOF, etc.	AUC ≥ 0.90 for 25 CpG sites	Illumina HumanMethylation450 Bead Chip
Bahado-Singh et al. [20]	Maternal peripheral blood (cell-free DNA)	12 isolated, non-syndromic CHD	Mid-Late pregnancy	cg04761177, cg21431091, cg01263077, etc.	Best AI model AUC = 0.97 (95% CI, 0.87-1.0)	Illumina Methylation EPIC Bead Chip
Zhou et al. [24]	Fetal cardiac tissue	17 isolated cardiac defects; 14 non-isolated cardiac defects	post-termination, 22–27 weeks	EGFR (intergenic), SLC19A1 (intergenic), NOTCH1 (intragenic)	EGFR intergenic region(*P* = 0.006); SLC19A1 intergenic region(*P* < 0.0001)	MeDIP-chip, Mass ARRAY EpiTYPER
Wijnands et al. [19]	Child peripheral blood leukocytes	84 (isolated pVSD)	Childhood (mean age 17 months)	cg17001566 (PRDM16 gene)	Methylation difference 4.84% in cases (*P* = 9.17 × 10^−8^)	Illumina HumanMethylation450 Bead Chip

## Methods

### Enrollment of participants and collection of amniotic fluid samples

The 159 pregnant women who underwent prenatal diagnosis during the second trimester were recruited from Women and Children’s Hospital, School of Medicine, Xiamen University. The phenotype and classification (simple congenital heart disease (SCHD) or complex congenital heart disease (CCHD)) was diagnosed by sonographers, pathologists, and pediatricians through systematic ultrasound, autopsy, or postnatal follow-up [25, 26]. Patients with amniotic cavity infection, hypertension, diabetes mellitus, severe anemia, hyperthyroidism or other chronic diseases were excluded. Controls were defined as fetuses without any congenital anomalies and were frequently matched to cases in terms of the mother’s age and the fetus’s sex. The study was conducted in accordance with the Declaration of Helsinki and was approved by the Ethics Committee of Human Research of Women and Children’s Hospital, School of Medicine, Xiamen University (ID: KY-2019-065), and all participants provided informed consent. Amniotic fluid samples were obtained when the subjects needed to undergo amniocentesis for medical reasons, with 5 mL of fluid collected with the participant’s consent. The samples were then centrifuged at 800 × g and 4 °C for 10 min, and the sediment and the supernatant were stored separately at -80 °C until analysis. The demographics and clinical characteristics of all participants, including maternal age, gestational age at sampling, fetal sex, maternal preexisting medical conditions and in-vitro fertilization (IVF), were also carefully registered.

## Whole-genome bisulfite sequencing of amniotic fluid

Whole-genome bisulfite sequencing libraries were constructed using the Acegen Single Strand Bisulfite-Seq Library Prep Kit (Acegen, Cat. No. AG0312) according to the manufacturer’s protocol. Briefly, sediment DNA was extracted with a TIANamp Genomic DNA Kit (Cat#DP304-03) according to the manufacturer’s instructions, and more than 0.5 µg of genomic DNA spiked with 1‰ unmethylated lambda DNA was fragmented to an average size of approximately 100–500 bp by sonication and treated with bisulfite. The heat-denatured DNA is subsequently transformed into a single strand ligated to the 3’-dA tail and tail. Next, the single-stranded DNA linked to the 3’ adapter is extended to a double strand and the 5-methylcytosine-modified adapters are ligated. Finally, the DNA was amplified with approximately 10 cycles of PCR using Illumina 8-bp dual index primers. The constructed WGBS libraries were then analyzed by Agilent 2100 Bioanalyzer and finally sequenced using a 150 × 2 paired-end sequencing protocol. DMR calling was performed with the binary segmentation algorithm using the software Matilene (version 0.2-8). The analysis was conducted with the following parameters: the minimum number of CpGs per region (-m) was set to 5, and the maximum distance between adjacent CpGs within a region (-d) was set to 200 bp. For each initially segmented region, differential methylation was assessed by jointly applying the Mann-Whitney U test and the two-dimensional Kolmogorov-Smirnov test. The *P*-values were then adjusted for multiple testing specifically within each of these algorithm-defined regions using the Benjamini-Hochberg false discovery rate (FDR) method.

## Gene ontology (GO) and Kyoto encyclopedia of genes and genomes (KEGG) analyses

To clarify the biological functions of the genes and the involved signaling pathways, we annotated each gene on the basis of the GO and KEGG databases. Enrichment calculations were performed using Fisher’s exact test. Furthermore, we also conducted GO and pathway enrichment analyses of the genes. Specifically, annotation mapping of the differentially expressed genes in the GO and KEGG database entries was performed, the number of genes in each GO and pathway entry was calculated, and a hypergeometric test was subsequently performed for statistical analysis. The GO and KEGG terms that were significantly enriched in the DEGs were selected. After the calculated *P* value was corrected by multiple hypothesis tests, a *P* value of 0.05 was taken as the threshold, and the GO and KEGG terms meeting this criterion were defined as the GO and KEGG terms significantly enriched in the target genes. The biological processes of GO and KEGG pathway enrichment analyses were carried out via the cluster Profiler package of R 4.0.0. The figure was drawn with ggplot2.

## Selection of candidate methylation markers

Candidate differentially methylated regions were selected according to group differences, adjusted *P* values, sequencing depths, correlations with cardiac development, and our previous metabolomics results. Fifty-two methylation regions were obtained. After removing methylation regions that were difficult to design with primers, we ultimately selected these candidate methylation markers for further validation.

## Targeted bisulfite sequencing

The DNA methylation level was analysed via MethylTarget^®^ (Genesky Biotechnologies Inc., Shanghai, China), an NGS-based multiple-targeted CpG methylation analysis method. Specifically, the genomic regions of interest were analysed and transformed to bisulfite-converted sequences via geneCpG software. The PCR primer sets were designed with Methylation Primer software from bisulfate-converted DNA. The DNA input for library preparation ranged from 200 to 800 ng. This quantity reliably yielded high-quality libraries with robust amplification efficiency in the laboratory workflow. Genomic DNA (400 ng) was subjected to sodium bisulfite treatment via the EZ DNA Methylation™-GOLD Kit (Zymo Research) according to the manufacturer’s protocols. Multiplex PCR was performed with optimized primer set combinations. A 20 µl PCR mixture was prepared for each reaction and included 1x reaction buffer (Takara), 3 mM Mg^2+^, 0.2 mM dNTPs, 0.1 µM each primer, 1 U of HotStarTaq polymerase (Takara) and 2 µl of template DNA. The PCR amplicons were diluted and amplified via indexed primers. Specifically, a 20 µl mixture was prepared for each reaction and included 1x reaction buffer (NEB Q5TM), 0.3 mM dNTPs, 0.3 µM F primer, 0.3 µM index primer, 1 U of Q5TM DNA polymerase (NEB) and 1 µL of diluted template. The PCR amplicons (170–270 bp) were separated via agarose electrophoresis and purified via a QIAquick Gel Extraction Kit (QIAGEN). Libraries from different samples were quantified and pooled together, followed by sequencing on the Illumina HiSeq platform according to the manufacturer’s protocols. Sequencing was performed in 2 × 150 bp paired-end mode with high sequencing depth (1000×). Fast Length Adjustment of SHort reads was used to merge paired-end reads. The fastq to fasta format step was then processed via the Fastx toolkit. Reads in fasta format were mapped to the targeted bisulfite genome (hg38) via BLAST. Unmapped reads were filtered, and mapped reads with coverage greater than 90% and identity greater than 95% were retained as effective reads and used for subsequent statistical analysis. The sequencing depth for each amplicon per sample was calculated by blasting the effective reads against the targeted genome region. Reads less than 10-fold were removed, and the overall sequencing depth for each sample was evaluated. Methylation and haplotypes were analysed via Perl script. Statistical analysis was performed via *t* tests and one-way analysis of variance (ANOVA). Given that the methylation analysis was focused on target regions, the *P*-values for differential methylation were adjusted for multiple testing using the Benjamini-Hochberg false discovery rate (FDR) correction applied across all tested CpG sites within the targeted region.

### Statistical analysis

The data were analysed via GraphPad Prism (version 9.0) and SPSS (version 29.0). Differences in the frequencies of these demographic characteristics between cases and controls were assessed using the chi-square test or Student’s t-test. A value of *P* < 0.05 was considered to indicate statistical significance. The positive predictive value (PPV) and the negative predictive value (NPV) are calculated based on the CHD prevalence of the respective cohorts. For methylation analysis, Benjamini-Hochberg false discovery rate (FDR) correction-*P* values of less than 0.05 were considered statistically significant.

## Results

### Study design and participants

After applying the inclusion and exclusion criteria to subjects with qualified biological samples, 135 amniotic fluid samples (80 CHD patients and 55 controls) were ultimately included in the present study. The detailed reasons for exclusion are provided in Supplementary Table 1. As listed in Table 2, the CHDs and Controls were well-matched in terms of demographic and clinical characteristics. Aside from a significant difference in the sampling gestational time, all other baseline variables, including maternal age and fetal gender, were comparable between the two groups. Notably, despite this difference in timing, all sample collections occurred within the second-trimester window. The subtype distributions of the 80 CHD patients are listed in Supplementary Table 2.

**Table 2:** Comparison of demographic characteristics between CHDs and Controls

Number of samples	CHDs	Controls	χ2/T	*P* value
	80	55		
Age, mean (95% CI)	31.13 (30.18–32.07)	32.65 (31.25–34.06)	1.872	0.064
Gestational age at sampling, mean (95%CI)	21.40 (20.62–22.18)	17.76 (17.37–18.16)	7.288	0.0001
Fetal gender, n (%)				
Male	43 (53.75)	30 (54.55)	0.008	0.927
Female	37 (46.25)	25 (45.45)		
Gravidity times, n (%)				
1	34 (42.50)	19 (34.55)	0.352	0.865
> 1	46 (57.50)	36 (65.45)		
Parity times, n (%)				
0	41 (51.25)	19 (34.55)	3.683	0.055
> 0	39 (48.75)	36 (65.45)		
Hypertension, n (%)				
Yes	1 (1.25)	0 (0)	0.693	0.405
No	79 (98.75)	55 (100)		
Diabetes, n (%)				
Yes	7 (8.75)	3 (5.45)	0.516	0.473
No	73 (91.25)	52 (94.55)		
In-vitro fertilization (IVF), n (%)				
Yes	12 (15)	4 (7.27)	1.863	0.172
No	68 (85)	51 (92.73)		

**Fig. 1:**
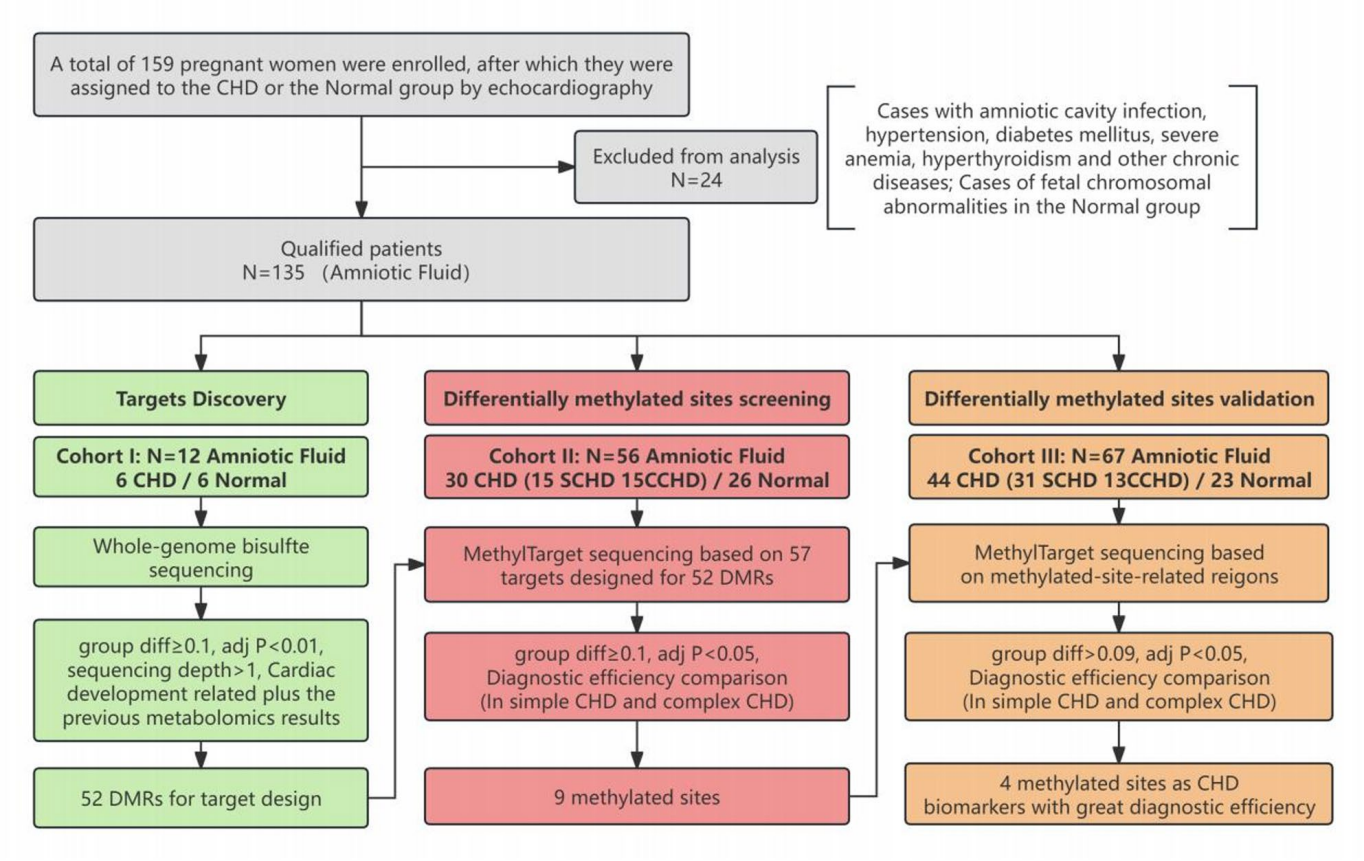
Workflow chart of the study design. CHD: congenital heart disease; DMR: differentially methylated region; MethylTarget: a targeted bisulfite sequencing method

An overview of the study design is shown in Fig. 1. In the discovery phase, we performed a genome-wide DNA methylation profiling assay in an independent cohort including 6 CHD patients and 6 normal controls (cohort I, *n* = 12), aiming to establish a DNA methylation landscape and identify CHD-specific methylation markers in amniotic fluid. The identified methylation markers that can be used for CHD diagnosis were subsequently validated in cohort II (*n* = 56, 15 SCHD, 15 CCHD and 26 normal controls). Finally, independent cohort III (*n* = 67, 31 SCHD, 13 CCHD and 23 normal controls) was used to evaluate the diagnostic efficacy of the validated biomarker panel for CHD.

### Genome-wide methylation analysis of amniotic fluid DNA reveals the methylation landscape for CHD

**Fig. 2:**
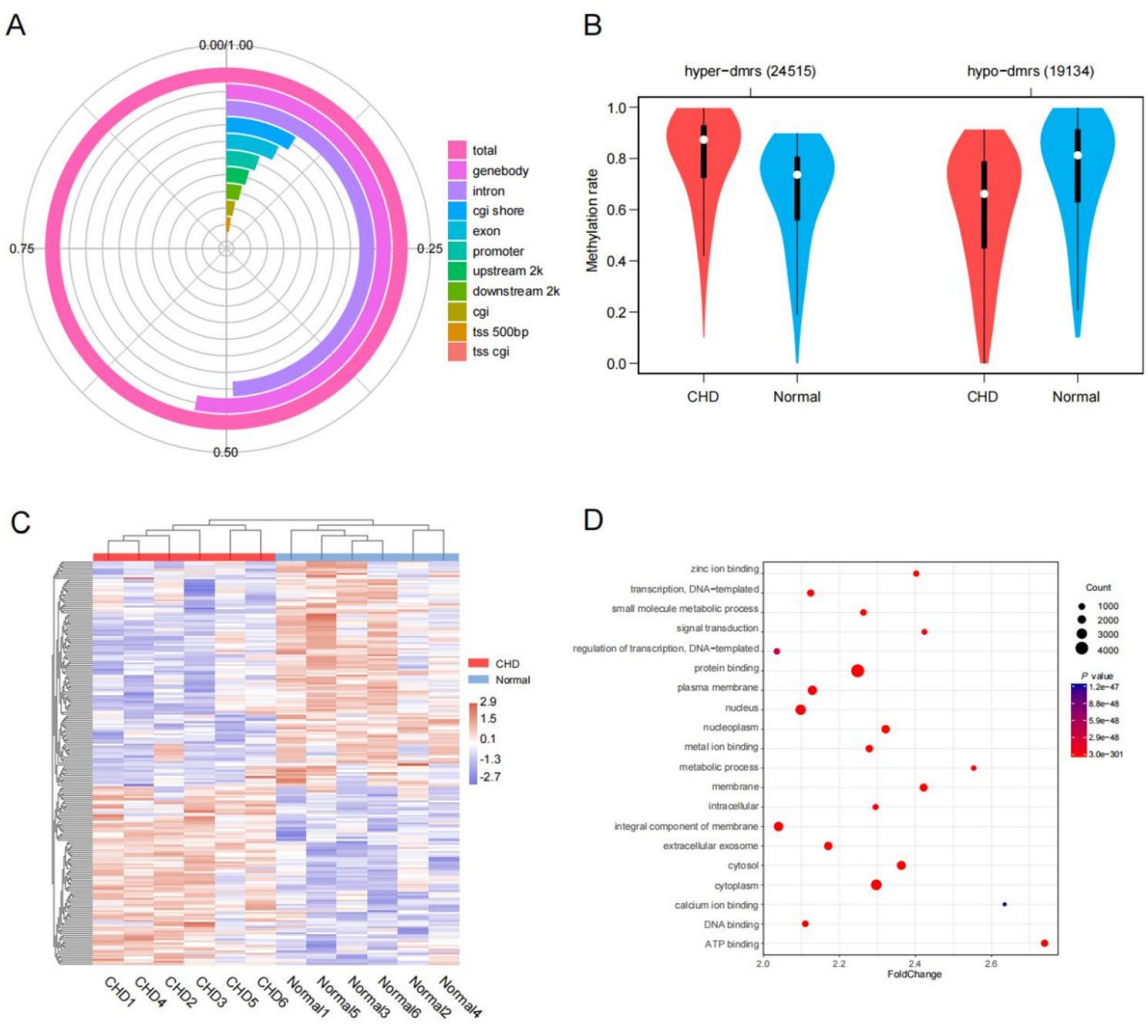
Whole-genome landscapes of DNA methylation signatures in the amniotic fluid of cohort Ⅰ. **A** Percentages of the DMR distribution in different genomic components, **B** violin plots for the methylation level and distribution of hyper and hypo-DMRs between the CHD and normal groups, **C** unsupervised hierarchical clustering with the top 200 DMRs; the methylation levels were normalized by the Z score, **D** the top 20 terms of the Gene Ontology enrichment results of DMRs between the CHD and normal groups

To evaluate whether any epigenetic alterations are associated with CHD pathogenesis, amniotic fluid samples from six CHD patients and six healthy controls during the second trimester were examined via WGBS. Following quality control and data preprocessing, the bisulfite conversion rates were all above 99%. The average sequencing depth for all WGBS samples was 8.04×, with 78.8% of CpG sites covered at ≥ 5×. However, no significant global DNA methylation alterations were noted between the CHD and control samples. The quality control information was shown in the Supplementary Table 3. On the basis of these findings, differentially methylated region (DMR) analysis was performed to identify epigenetic differences between the two groups. A total of 43,649 DMRs (24,515 hypermethylated and 19,134 hypomethylated) were identified in the CHD samples relative to the control samples, 2412 of which (5.52%) were located in promoter regions (Fig. 2A and B). By using the top 200 DMRs (Supplementary Table 4), the samples were separated into two groups corresponding to CHD patients and controls (Fig. 2C), which revealed distinct DNA methylation profiles in the amniotic fluid between the two groups. These results highlight the potential of amniotic fluid methylation patterns to serve as biomarkers for prenatal CHD diagnosis.

To characterize the functional relevance of these DMRs, all DMRs were associated with their nearest gene, and Gene Ontology (GO) and pathway enrichment analyses were performed (Supplementary Tables 5, 6). The DMRs were found to be significantly enriched in the cellular component category (9 of the top 20 terms according to the Gene Ontology results) (Fig. 2D). Moreover, the MAPK, PI3K-Akt and calcium signaling pathways were also significantly enriched in DMRs according to the KEGG analysis (Supplementary Table 6). The abnormal regulation of these pathways is involved in vascular remodelling and abnormal heart development [27–29].

### Identification of DNA methylation markers in maternal amniotic fluid to distinguish CHD patients from normal controls

After identifying many DMRs in the discovery phase, we applied a series of screening principles to determine the most important and specific DNA methylation markers for CHD. The specific screening criteria for candidate DMRs in WGBS are as follows: (**i**) stringent statistical thresholds (adjusted *P* value < 0.01, group diff > 0.1 and sequencing depth ≥ 5×); (**ii**) genes that have been reported in existing studies to be directly related to heart development; or (**iii**) DMRs related to the key metabolic pathways identified in our previous metabolomics analysis of these amniotic fluid samples.

**Fig. 3:**
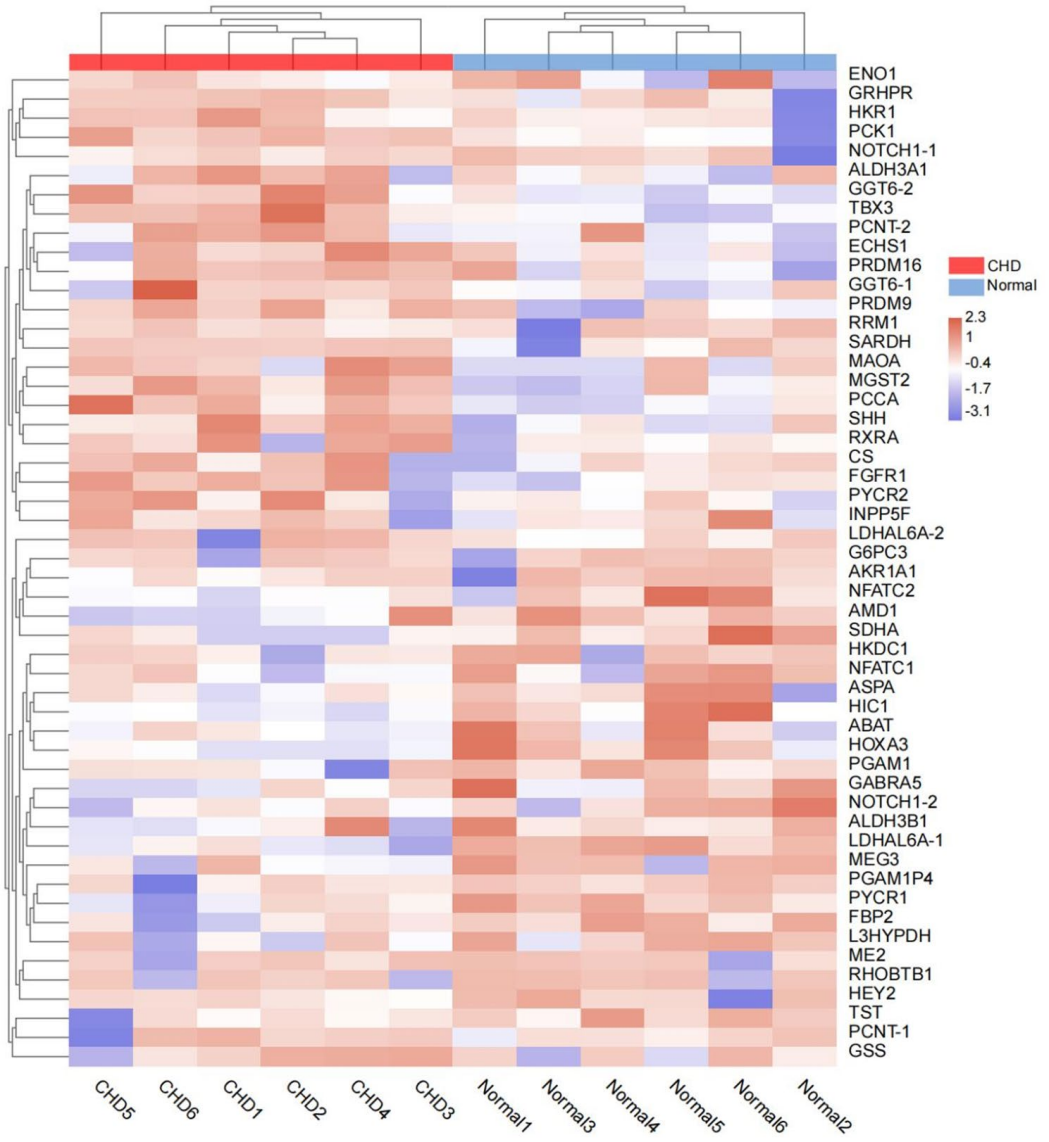
Unsupervised hierarchical clustering of cohort I on the basis of 52 candidate DMRs. Methylation levels were normalized by the Z score. Only the nearest-protein coding genes of DMRs are labelled in the figure, and numbers were added after duplicate gene names for discrimination (the detailed information of candidate DMRs is shown in Table 3)

**Fig. 4:**
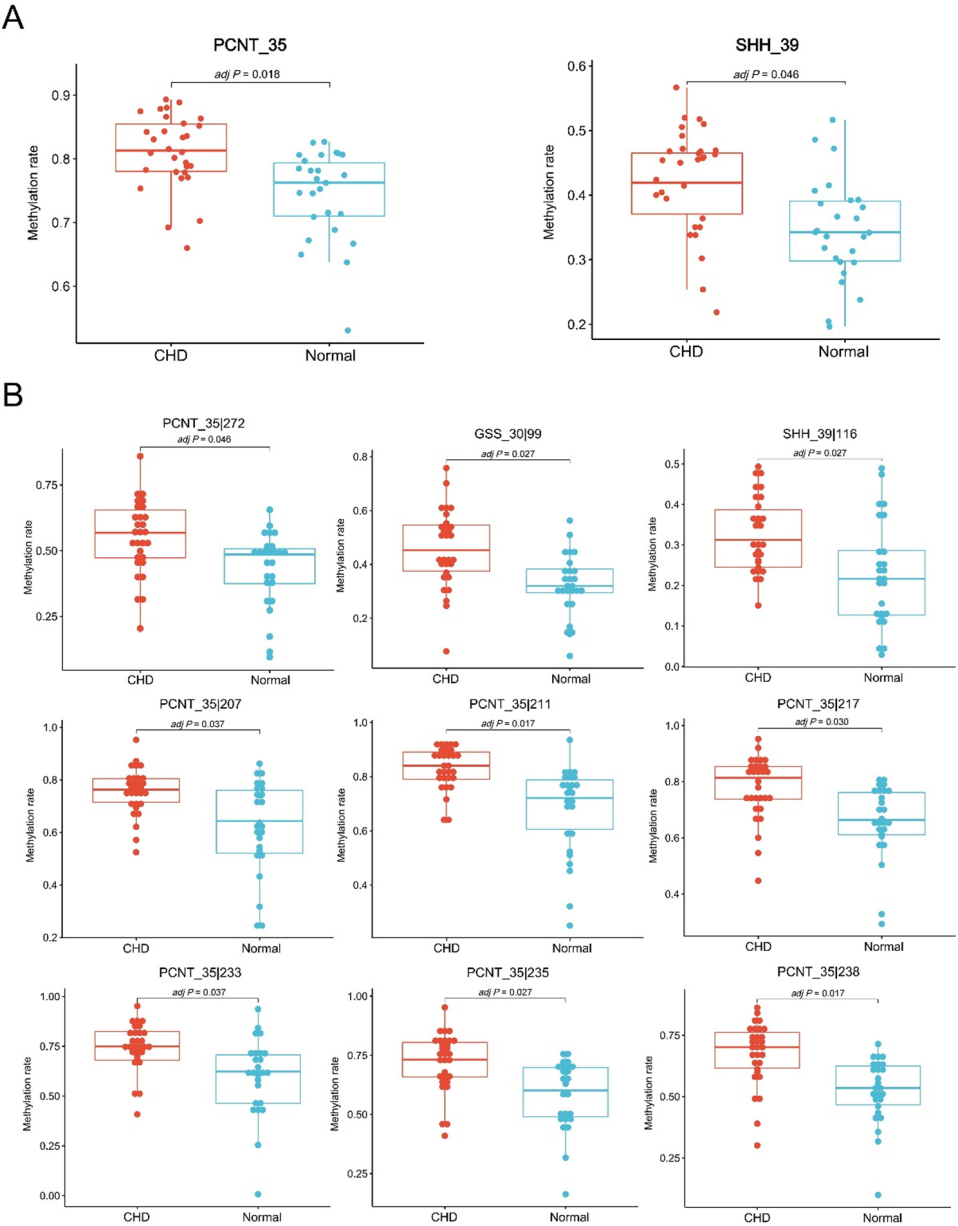
Box plots of differentially methylated targets (**A**) or sites (**B**) of cohort II. *P* values were determined by a *t* test and then adjusted using the Benjamini-Hochberg method (*n* = 56)

We identified 52 DMRs as candidate biomarkers for subsequent validation (Table 3). By using these candidate DMRs, the 12 samples of the discovery phase can also be separated into two groups corresponding to CHD patients and controls (Fig. 3), thus suggesting that these DMRs may be potential biomarkers for CHD. After removing 14 DMRs that were difficult to design with primers, we designed 57 pairs of primers (Supplementary Table 7) to cover the remaining 38 candidate DMRs and validated them through TBS in a new independent Cohort II. The results showed that TBS achieved a very high average depth of approximately 5.21E + 03 per base, and 100% of CpG sites had a mean coverage ≥ 10× across samples, ensuring robust methylation calls. The bisulfite conversion efficiency for all samples was consistently > 99%, confirming high conversion rates. As demonstrated in Fig. 4A, only two target regions (*PCNT_35* and *SHH_39*) exhibited statistically significant differences in methylation levels between CHD patients and normal controls. In addition, 25 differentially methylated sites were identified between the two groups (Table 4). To facilitate subsequent clinical application in prenatal CHD diagnosis, we exclusively selected hypermethylated sites in CHD cases with group differences ≥ 0.1 for further evaluation and validation. Ultimately, nine differentially methylated sites were retained for further evaluation, with seven sites (77.8%) surprisingly originating from the two differentially methylated target regions *PCNT_35* and *SHH_39* (Fig. 4B). The specific results of the TBS are shown in Supplementary Table 8. *PCNT* and *SHH* are directly involved in the regulation of cellular proliferation [30, 31]. These results further underscore the diagnostic potential of amniotic fluid-derived DNA methylation biomarkers for CHD and suggest a pathogenetic link between cell proliferation and CHD development.

**Table 3:** The differential methylation regions (DMRs) chosen to be validated as CHD biomarkers

DMRNo.	Genomic position (hg38)	Nearest-protein coding gene	Located component	CHD mean methylation (%)	Normal mean methylation (%)	Adjust *P*-value*	Screening factors a: Cardiac-development related b: Candidate key metabolic pathway
1	chr5: 217,375–217,525	*SDHA*	Promoter	9.98	25.59	3.86E−03	b (KEGG Terms: hsa00020)
2	chr17: 3,476,037–3,476,300	*ASPA*	Promoter	38.73	58.63	3.79E−03	b (KEGG Terms: hsa00250)
3	chr11: 4,136,087–4,136,179	*RRM1*	Promoter	78.89	95.84	3.97E−04	b (KEGG Terms: hsa00480)
4	chr17: 4,556,542–4,557,050	*GGT6*	Exon	80.87	69.52	2.39E−03	b (KEGG Terms: hsa00480)
5	chr17: 4,562,019 − 4,561,788	*GGT6*	Promoter	62.38	45.94	1.03E−02	b (KEGG Terms: hsa00480)
6	chr16: 8,719,107–8,719,173	*ABAT*	Promoter	10.54	26.80	4.33E−03	b (KEGG Terms: hsa00250)
7	chr1: 8,880,615–8,880,837	*ENO1*	Promoter	33.32	53.55	1.11E−02	b (KEGG Terms: hsa00010)
8	chr3: 9,348,822–9,349,243	*PGAM1P4*	Promoter	82.33	92.83	1.13E−03	b (KEGG Terms: hsa00010)
9	chr11: 18,455,050 − 18,454,832	*LDHAL6A*	Promoter	77.01	90.03	5.42E−05	b (KEGG Terms: hsa00010) b (KEGG Terms: hsa00270) b (KEGG Terms: hsa00620)
10	chr11: 18,456,209–18,456,496	*LDHAL6A*	Promoter	88.81	68.57	4.10E−03	b (KEGG Terms: hsa00010) b (KEGG Terms: hsa00270) b (KEGG Terms: hsa00620)
11	chr17: 19,745,979–19,746,341	*ALDH3A1*	Promoter	48.80	33.63	4.33E−03	b (KEGG Terms: hsa00010)
12	chr20: 34,956,912–34,957,140	*GSS*	Promoter	34.62	24.44	1.01E−02	b (KEGG Terms: hsa00480)
13	chr22: 37,021,909 − 37,021,667	*TST*	Promoter	56.39	75.31	6.31E−03	b (KEGG Terms: hsa00270)
14	chr9: 37,420,545–37,420,714	*GRHPR*	Promoter	86.60	76.08	8.73E−03	b (KEGG Terms: hsa00260) b (KEGG Terms: hsa00620) b (KEGG Terms: hsa00630)
15	chr17: 44,068,819–44,068,982	*G6PC3*	Promoter	77.06	87.86	7.99E−03	b (KEGG Terms: hsa00010)
16	chrX: 43,654,966–43,655,257	*MAOA*	Promoter	47.76	20.63	5.89E−03	b (KEGG Terms: hsa00260) b (KEGG Terms: hsa00330)
17	chr1: 45,549,083–45,549,108	*AKR1A1*	Promoter	80.26	93.97	3.48E−03	b (KEGG Terms: hsa00010)
18	chr18: 50,877,248–50,877,422	*ME2*	Promoter	75.01	85.09	1.33E−02	b (KEGG Terms: hsa00620)
19	chr20: 57,559,057–57,559,280	*PCK1*	Promoter	63.43	47.94	1.74E−03	b (KEGG Terms: hsa00010) b (KEGG Terms: hsa00020) b (KEGG Terms: hsa00620)
20	chr12: 56,301,713 − 56,301,449	*CS*	Promoter	69.65	49.14	7.93E−04	b (KEGG Terms: hsa00020) b (KEGG Terms: hsa00630)
21	chr14: 59,485,854 − 59,485,642	*L3HYPDH*	Promoter	79.49	91.60	1.11E−02	b (KEGG Terms: hsa00330)
22	chr11: 68,009,134 − 68,008,871	*ALDH3B1*	Promoter	27.54	56.94	6.73E−04	b (KEGG Terms: hsa00010)
23	chr10: 69,220,483–69,220,766	*HKDC1*	Promoter	46.86	64.13	1.57E−04	b (KEGG Terms: hsa00010)
24	chr17: 81,936,476–81,936,797	*PYCR1*	Promoter	21.69	39.31	3.02E−03	b (KEGG Terms: hsa00330)
25	chr9: 94,595,029–94,595,094	*FBP2*	Promoter	51.21	68.35	1.95E−04	b (KEGG Terms: hsa00010)
26	chr10: 97,425,166–97,425,435	*PGAM1*	Promoter	58.79	76.20	1.16E−03	b (KEGG Terms: hsa00010) b (KEGG Terms: hsa00260)
27	chr13: 100,088,285–100,088,408	*PCCA*	Promoter	74.14	58.87	7.55E−03	b (KEGG Terms: hsa00630)
28	chr6: 110,873,350–110,873,452	*AMD1*	Promoter	45.51	65.89	4.10E−03	b (KEGG Terms: hsa00270) b (KEGG Terms: hsa00330)
29	chr10: 133,373,946–133,374,209	*ECHS1*	Promoter	36.44	19.67	1.40E−03	b (KEGG Terms: hsa00010)
30	chr9: 133,740,358–133,740,597	*SARDH*	Promoter	88.83	77.75	8.52E−03	b (KEGG Terms: hsa00260)
31	chr4: 139,665,181–139,665,246	*MGST2*	Promoter	66.53	49.91	7.04E−04	b (KEGG Terms: hsa00480)
32	chr1: 225,924,825–225,924,978	*PYCR2*	Promoter	38.31	23.08	8.88E−04	b (KEGG Terms: hsa00330)
33	chr5: 23,507,291–23,507,310	*PRDM9*	Promoter	82.95	56.07	1.78E−03	a [32]
34	chr19: 37,316,620–37,316,919	*HKR1*	Promoter	67.32	47.80	8.85E−05	a [16]
35	chr12: 114,680,732–114,681,052	*TBX3*	Exon/intron	43.35	24.32	6.98E−05	a [32]
36	chr1: 3,405,292–3,405,502	*PRDM16*	Exon/intron	48.59	29.78	2.29E−07	a [19]
37	chr7: 155,813,710–155,813,983	*SHH*	Promoter	62.84	45.53	5.42E−05	a [33]
38	chr21: 46,409,720–46,409,976	*PCNT*	Intron	96.80	81.00	1.25E−03	a [30]
39	chr21: 46,409,907–46,410,206	*PCNT*	Intron	96.10	83.66	4.63E−06	a [30]
40	chr9: 134,360,406 − 134,360,162	*RXRA*	Intron	45.55	33.12	2.28E−04	a [34]
41	chr8: 38,465,624–38,465,851	*FGFR1*	Intron	14.30	3.20	1.28E−05	a [35]
42	chr10: 60,945,945–60,946,039	*RHOBTB1*	Promoter	85.82	95.89	5.56E−04	a [36]
43	chr7: 27,110,446–27,110,606	*HOXA3*	Exon	0.42	12.61	5.75E−05	a [37]
44	chr18: 79,485,086–79,485,433	*NFATC1*	Intron	15.66	28.41	9.02E−04	a [37]
45	chr6: 125,759,416 − 125,759,157	*HEY2*	Exon	67.66	81.20	4.82E−04	a [32]
46	chr17: 2,058,321–2,058,362	*HIC1*	Exon	11.13	25.88	4.82E−07	a [9]
47	chr15: 26,892,396–26,892,458	*GABRA5*	Intron	5.86	22.27	3.97E−04	a [35]
48	chr14: 100,826,144–100,826,217	*MEG3*	Promoter	29.83	52.85	8.98E−07	a [38]
49	chr20: 51,492,398–51,492,428	*NFATC2*	Intron	20.77	47.61	3.97E−04	a [37]
50	chr9: 136,516,459–136,516,693	*NOTCH1*	Intron	71.99	84.93	4.02E−04	a [39]
51	chr9: 136,533,583 − 136,533,372	*NOTCH1*	Exon	41.84	72.10	1.89E−05	a [39]
52	chr10: 119,818,816–119,819,049	*INPP5F*	Promoter	57.78	43.50	1.34E−03	a [40]

*The *P-*value were adjusted using the Benjamini-Hochberg method

**Table 4:** Features of 25 differential methylation sites of the cohort II

Methylated marker*	CpG site (hg38)	Located gene	Located component	Group difference**	*P*-value	Adjust *P*-value***
PCNT_35|211	chr 21: 46,410,117	PCNT	Intron	0.157	7.23E−05	1.73E−02
PCNT_35|233	chr 21: 46,410,139	PCNT	Intron	0.152	1.21E−03	3.72E−02
PCNT_35|238	chr 21: 46,410,144	PCNT	Intron	0.149	1.15E−04	1.73E−02
PCNT_35|235	chr 21: 46,410,141	PCNT	Intron	0.140	3.40E−04	2.70E−02
PCNT_35|207	chr 21: 46,410,113	PCNT	Intron	0.131	1.34E−03	3.72E−02
GSS_30|99	chr 20: 34,957,010	GSS	Promoter	0.130	3.94E−04	2.70E−02
PCNT_35|272	chr 21: 46,410,178	PCNT	Intron	0.124	2.07E−03	4.64E−02
PCNT_35|217	chr 21: 46,410,123	PCNT	Intron	0.123	5.83E−04	2.96E−02
SHH_39|116	chr 7: 155,813,825	ECHS1	Promoter	0.107	3.33E−04	2.70E−02
ECHS1_8|199	chr 10: 133,374,144	ECHS1	Promoter	0.094	5.46E−04	2.96E−02
ECHS1_8|231	chr 10: 133,374,176	ECHS1	Promoter	0.091	1.43E−03	3.72E−02
NOTCH1_56|169	chr 9: 136,533,751	NOTCH1	Exon	0.086	1.42E−03	3.72E−02
RXRA_47|192	chr 9: 134,360,597	RXRA	Intron	0.085	1.26E−03	3.72E−02
RXRA_47|123	chr 9: 134,360,528	RXRA	Intron	0.083	1.08E−03	3.72E−02
RXRA_47|106	chr 9: 134,360,511	RXRA	Intron	0.080	1.17E−03	3.72E−-02
SARDH_46|134	chr 9: 133,740,491	SARDH	Promoter	−0.029	1.02E−03	3.72E−02
HEY2_38|67	chr 6: 125,759,482	HEY2	Exon	−0.038	1.37E−03	3.72E−02
HOXA3_42|21	chr 7: 27,110,466	HOXA3	Exon	−0.050	1.47E−03	3.72E−02
HOXA3_42|72	chr 7: 27,110,517	HOXA3	Exon	−0.051	1.24E−04	1.73E−02
HOXA3_42|66	chr 7: 27,110,511	HOXA3	Exon	−0.053	9.43E−04	3.72E−02
HEY2_38|70	chr 6: 125,759,485	HEY2	Exon	−0.055	4.35E−04	2.70E−02
HOXA3_42|56	chr 7: 27,110,501	HOXA3	Exon	−0.056	1.64E−03	3.98E−02
PCK1_32|158	chr 20: 57,559,214	PCK1	Promoter	−0.073	1.82E−04	2.03E−02
HKDC1_4|31	chr 10: 69,220,513	HKDC1	Promoter	−0.093	2.08E−03	4.64E−02
NOTCH1_52|109	chr 9:136516567	NOTCH1	Intron	−0.162	7.63E−05	1.73E−02

*Methylated marker: The code name of the differentially methylated sites in this study

**The Group Difference represents the difference in mean methylation values (range 0–1, representing 0-100% methylation) between the CHD and control groups

***The *P-*value were adjusted using the Benjamini-Hochberg method

### Performance of the selected methylation markers in another independent cohort

**Fig. 5:**
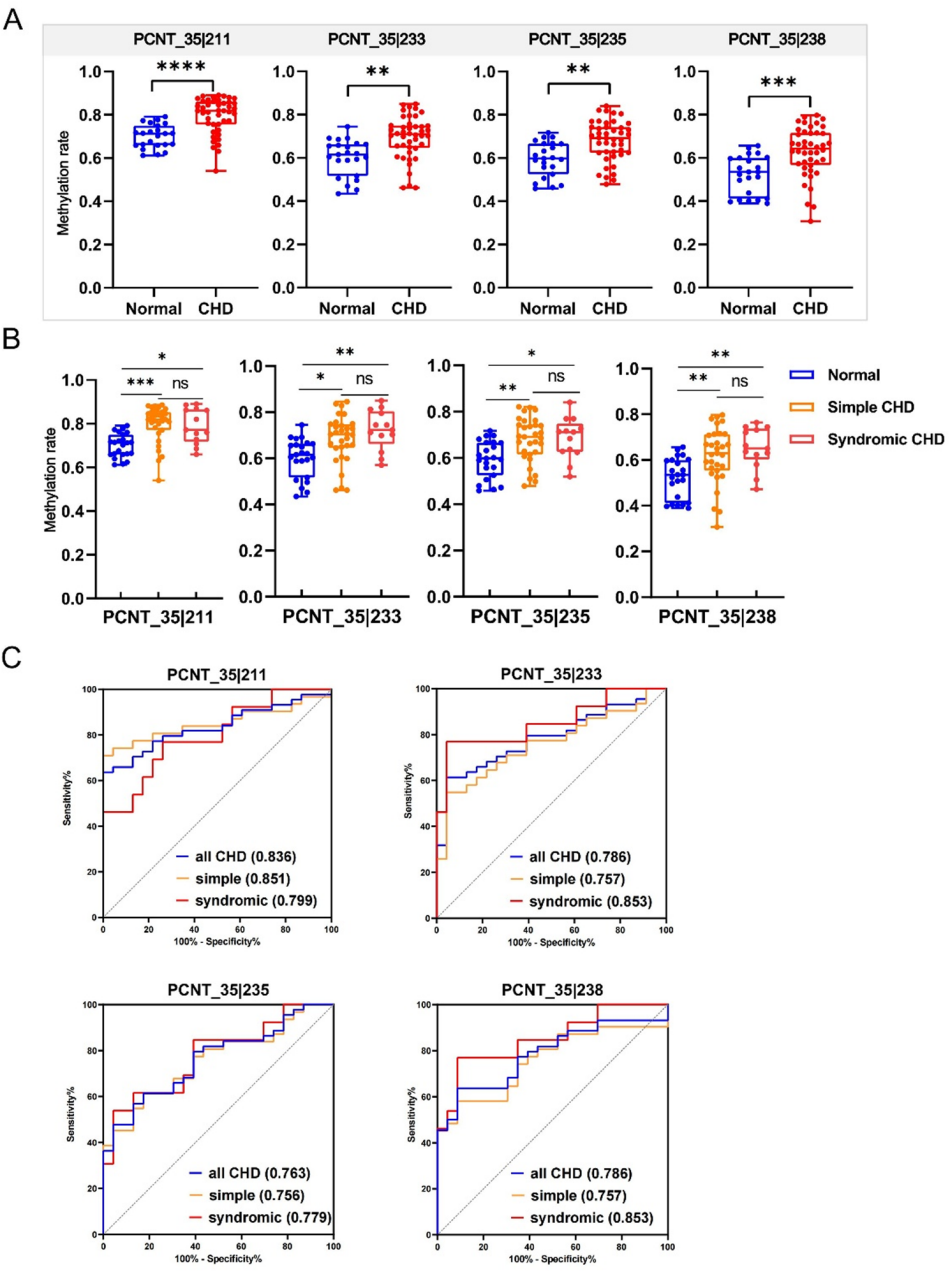
Identification and validation of CHD-methylation markers in amniotic fluid. **A** Box plots showing the methylation rates of four methylation markers validated by the TBS in amniotic fluid samples, **B** methylation rates of four methylation markers in 23 normal controls, 31 simple CHD patients and 13 complex CHD patients in the TBS, **C** ROC analysis of the sensitivity and specificity of the four methylation markers in the prediction of CHD in patients in validation cohort III. The AUCs for the different categories (all, simple or complex CHD) are shown in the legend (*n* = 67). *P* values were determined by a *t* test and then adjusted using the Benjamini-Hochberg method, *adj *P* ≤ 0.05; **adj *P* ≤ 0.01; ***adj *P* ≤ 0.001; ****adj *P* ≤ 0.0001; ns, no significance

Based on the results of cohort II, we have identified 9 hypermethylated sites. Considering the future development plan of PCR methods, we selected the four markers with the highest group differences in the cohort II to construct the predictive model, namely *PCNT|35_211*,* PCNT|35_233*,* PCNT|35_238*,* PCNT|35_235*. We next validated the DMRs and associated region previously identified in the cohort II—*PCNT_35*, *SHH_39* and *GSS_30*—in an independent cohort III. Their methylation levels were measured using TBS to evaluate the diagnostic efficacy and generalizability of above markers. Moreover, we also conducted TBS of the CpG islands in the promoter regions of *PCNT* and *SHH* (Supplementary Table 9). Consistent with the findings in the cohort II, *PCNT_35* remained a significantly differentially methylated region in the cohort III. Furthermore, a total of 11 differentially methylated sites were identified in the cohort III. Of these, 8 sites had also been identified as differentially methylated in the cohort II. The four candidate methylation markers still showed significant hypermethylation in the CHD group of the cohort III, and the differences in methylation levels and detailed information are shown in Fig. 5A; Table 5.

**Table 5:** Features of 11 differential methylation sites of the cohort III

Methylated marker*	CpG site (hg38)	Located gene	Located component	Group difference**	*P*-value	Adjust *P*-value***
GSS_30|99	chr 20: 34,957,010	GSS	Promoter	0.121	5.945E−05	8.125E−04
PCNT_35|238	chr 21: 46,410,144	PCNT	Intron	0.107	8.827E−05	9.048E−04
PCNT_35|211	chr 21: 46,410,117	PCNT	Intron	0.096	3.143E−07	1.289E−05
PCNT_35|233	chr 21: 46,410,139	PCNT	Intron	0.094	1.574E−04	1.291E−03
PCNT_35|217	chr 21: 46,410,123	PCNT	Intron	0.093	1.167E−06	2.393E−05
PCNT_35|257	chr 21: 46,410,163	PCNT	Intron	0.089	4.712E−04	2.415E−03
PCNT_35|235	chr 21: 46,410,141	PCNT	Intron	0.086	2.722E−04	1.594E−03
PCNT_35|272	chr 21: 46,410,178	PCNT	Intron	0.078	9.888E−03	4.504E−02
GSS_30|127	chr 20: 34,957,038	GSS	Promoter	0.072	1.233E−02	4.878E−02
PCNT_35|207	chr 21: 46,410,113	PCNT	Intron	0.068	1.902E−04	1.299E−03
PCNT_35|170	chr 21: 46,410,076	PCNT	Intron	−0.025	1.309E−02	4.878E−02

*Methylated marker: The code name of the differentially methylated sites in this study

**The Group Difference represents the difference in mean methylation values (range 0–1, representing 0-100% methylation) between the CHD and control groups

***The *P-*value were adjusted using the Benjamini-Hochberg method

To analyse the CHD form specificity of the above candidate sites, we divided the CHD samples in cohort III into SCHD and CCHD groups. As shown in Fig. 5B, the methylation levels of 4 sites from *PCNT* were higher in SCHD or CCHD samples than in normal controls, and there was no obvious difference between SCHD and CCHD at each site. As shown in Fig. 5C, each methylation marker could distinguish SCHD and CCHD patients from normal controls in both sets.

The performance and generalization of the prediction model composed of four loci were validated in cohort III. The unadjusted cutoff values for each marker were referenced to its Youden index in the cohort II, as follows: *PCNT|35_211 (0.8022); PCNT|35_233 (0.7260); PCNT|35_238 (0.6605); and PCNT|35_235 (0.7279)*. The sample was defined as positive if any individual marker scored as methylated. The primary rationale for this design is to ensure full transparency, ease of clinical implementation and direct interpretability, where each component and the final decision logic are explicitly defined. Then, the diagnostic performance of the four-marker panel was evaluated in an independent validation cohort III. The panel achieved a sensitivity of 70.45% (95% CI: 54.8–83.2%) and a specificity of 95.65% (95% CI: 78.1–99.9%). Based on the observed prevalence of 65% in the validation cohort, the positive and negative predictive values were 96.9% (95% CI: 81.9–99.5%) and 62.9% (95% CI: 51.5–72.9%), respectively. Comparable diagnostic performance was also observed when the model was applied to cohort II and to the SCHD and CCHD sample sets separately. The detailed results of these analyses are summarized in Tables 6 and 7.

**Table 6:** The diagnostic efficacy of the prediction model composed of PCNT|35_211, PCNT|35_233, PCNT|35_238, and PCNT|35_235 in the cohort III or cohort II

Prediction model (PCNT|35_211, PCNT|35_233, PCNT|35_238, PCNT|35_235)	Cohort II			Cohort III		
	Actual results		Total	Actual results		Total
	Positive	Negative		Positive	Negative	
Positive	25	7	32	31	1	32
Negative	5	19	24	13	22	35
Total	30	26	56	44	23	67
Sensitivity (95% CI)	83.33% (65.3–94.4%)			70.45% (54.8–83.2%)		
Specificity (95% CI)	73.08% (52.2–88.4%)			95.65% (78.1–99.9%)		
Positive predictive value, PPV (95% CI)	78.1% (65.0–87.3%)			96.9% (81.9–99.5%)		
Negative predictive value, NPV (95% CI)	79.2% (62.3–89.7%)			62.9% (51.5–72.9%)		

**Table 7:** The diagnostic efficacy of the prediction model composed of PCNT|35_211, PCNT|35_233, PCNT|35_238, and PCNT|35_235 in SCHDs or CCHDs vs. all Controls

Prediction model (PCNT|35_211, PCNT|35_233, PCNT|35_238, PCNT|35_235)	SCHDs versus all controls			CCHDs versus all controls		
	Actual results		Total	Actual results		Total
	Positive	Negative		Positive	Negative	
Positive	35	8	43	21	8	29
Negative	11	41	52	7	41	48
Total	46	49	95	28	49	77
Sensitivity (95% CI)	76.09% (61.2–87.4%)			75.00% (55.1–89.3%)		
Specificity (95% CI)	83.67% (70.3–92.7%)			83.67% (70.3–92.7%)		
Positive predictive value, PPV (95% CI)	81.4% (69.5–89.4%)			72.4% (57.3–83.7%)		
Negative predictive value, NPV (95% CI)	78.8% (68.7–86.4%)			85.4% (75.3–91.8%)		

## Discussion

CHD is the birth defect with the highest incidence, and many children die or have serious sequelae worldwide [1]. Early fetal echocardiography has become an established tool for the detection of CHD, however, owing to factors such as tester expertise, equipment quality and the complex types of CHD, only approximately 40% of CHD cases are diagnosed by echocardiography [41]. Therefore, it is urgent to improve the clinical diagnosis of CHD. However, the underlying pathogenesis of CHD remains poorly understood, and only approximately 20% of CHD incidence can be attributed to genetic syndromes, maternal diabetes or teratogen exposure [42]. Advances in epigenetic research have increasingly established the association between DNA methylations during cardiac development and CHD [43]. Furthermore, amniotic fluid in the second trimester—composed primarily of fetal urine and exfoliated cells—serves as the immediate environment for fetal growth [21]. This relative simplicity makes it an ideal sample for assessing DNA methylation status in fetuses with CHD. However, owing to the limitations of sample sources, only a small amount of DNA methylation data from CHD fetal amniotic fluid are available [44].

In this study, we analyzed the amniotic fluid DNA methylation profile of CHD patients and found significant differences between CHD patients and normal controls. Then, we identified and validated a biomarker panel based on four methylation sites through WGBS and TBS. This biomarker panel has decent sensitivity of 70.45% (95% CI: 54.8–83.2%) and a specificity of 95.65% (95% CI: 78.1–99.9%) in cohort III for identifying patients with CHD and can be used to improve prenatal diagnosis. To our knowledge, this work reports the first validated amniotic-fluid methylation panel for CHD. Furthermore, our sample size is larger compared to other early, tissue-specific discovery studies in the field [16, 24], and the solid predictive accuracy we observed suggests that amniotic fluid is a valuable source for CHD biomarkers.

A meta-analysis encompassing 4,992 prenatal ultrasound cases of CHD revealed that screening for CHD by SAS in unselected populations yields a prenatal detection rate of approximately 45% [45]. A lack of adaptational skills and suboptimal cardiac view quality both appear to play important roles in the failure to detect CHD prenatally [46]. In contrast, methylation-based detection methods are independent of operator experience, eliminating subjective factors in ultrasound imaging and reducing reliance on experienced sonographer. Furthermore, metabolomics and proteomics have demonstrated significant potential in the prenatal diagnosis of CHD. A study based on maternal plasma [7] identified a panel of nine protein biomarkers that, through a machine learning model, achieved high diagnostic performance with an AUC of 0.964. Several other studies [8, 47] using different sample types have also revealed diagnostically relevant metabolites: in amniotic fluid, uric acid (AUC = 0.890) and novel metabolite combinations (AUC up to 0.988) showed good discriminative ability; in pediatric serum, taurine, glutamine, and glutamate (individual markers with AUC > 0.80) as well as the combination of betaine, taurine, glutamine, and phenylalanine (AUC = 0.949) were also found to have high diagnostic value for CHD [48].

In this study, our methylation biomarker panel likewise exhibited considerable diagnostic efficacy for CHD and remained stable across both simple and complex CHD subtypes. Notably, while many omics investigations remain at the discovery stage, this work has advanced into validation based on independent cohorts—representing a concrete step toward clinical application. Besides, the methylation biomarker panel in this study is cost-competitive with the conventional next generation sequencing. Due to the limited number of target loci (only 1 target region), the reduced sequencing data yield significantly lowers costs compared to CNV-seq or whole exome sequencing. Furthermore, compared to current clinical amniotic-fluid tests (such as karyotype analysis, CNV-seq and whole exome sequencing), this approach does not require specialized bioinformatics analysis workflows, resulting in shorter turnaround times. And further development into methylation-specific PCR is expected to reduce the turnaround time to within 24 h. However, it is important to acknowledge that the fetal echocardiography remains the widely recommended, first‑line, and authoritative method for prenatal CHD screening in routine antenatal care [49]. Our assay is intended to serve as a supplementary molecular tool that could aid in the interpretation of equivocal ultrasound findings or provide additional reference in high‑risk pregnant woman.

We identified 43,469 DMRs overall by comparing the whole-genome methylation results of the CHD patients and normal controls in cohort I, and cluster analysis of the top-DMRs distinguished CHD patients from normal controls. These findings suggest differential DNA methylation in amniotic fluid between CHD fetuses and healthy controls, highlighting the potential of amniotic fluid DNA methylation as a prenatal diagnostic biomarker for CHD. Through KEGG functional enrichment analysis, these DMRs were shown to be involved in the regulation of the actin cytoskeleton; adrenergic signalling in cardiomyocytes, and the PI3K-Akt, Rap1, and MAPK signalling pathways. In addition, DMRs were also significantly enriched in the oxytocin signaling pathway and its downstream calcium signaling pathway [50].

Dysregulation of these pathways has been extensively investigated in the context of cardiac development. Aberrant modulation of the MAPK pathway leads to Noonan syndrome-associated cardiac defects [14], whereas excessive melatonin supplementation interferes with embryonic heart development by inducing apoptosis and cell cycle arrest via the PI3K-Akt signaling pathway [51]. Multiple studies have revealed the critical role of the actin cytoskeleton in the development of the secondary heart field (SHF) in mice [52]. Finally, dysregulation of calcium activity results in failure in every step of cardiac development, including the differentiation of cardiac progenitor cells and cardiac tube formation [53].

Notably, even when analyzing placental samples, a methylation study of CHD-discordant monozygotic twins identified KEGG enrichment profiles congruent with our findings [17]. This intersample consistency implies the existence of similar abnormal DNA methylations and pathogenic mechanisms in near-environmental samples from CHD fetuses. These findings from the placental analysis suggest that the same epigenetic signature may also be detectable in cell-free DNA (cfDNA) from maternal peripheral blood. Moving forward, we aim to translate this panel by evaluating its applicability in this less invasive matrix, with the goal of meeting the clinical need for widespread noninvasive prenatal screening (NIPS) [54].

In this study, the methylation levels in the intronic region of *PCNT* exhibited consistent and statistically significant differences across both validation cohorts, with highly concordant differential methylation sites. Given that the major differential methylation sites are concentrated in the intron region of *PCNT* (chr21: 46,409,907 − 46,410,206, hg38), we therefore conducted a separate analysis of this region. Previous study [55] have reported that the functional enhancers of *PCNT* in human stem cells do not contain this intron region sequence. Therefore, we further analyzed the splicing regulatory potential of this region. Using the motif from the ATtRACT database for scanning, we found that this sequence matches the binding motifs of *SRSF2*,* SRSF3*, and *SRSF6* splicing factors of the SRSF family (*P* < 0.001), suggesting its potential as a splicing regulatory element. Study have shown that gene body methylation can influence splicing through various mechanisms, such as indirectly recruiting splicing factors of the SRSF family via the H3K9me3-HP1 pathway or modulating the elongation rate of RNA polymerase II through methyl-binding proteins like MeCP2 [56]. Therefore, we hypothesize that the hypermethylation in this intronic region may regulate the processing of *PCNT* mRNA by affecting such transcription-coupled splicing processes. Furthermore, a more general perspective holds that DNA methylation in gene bodies (including introns) is often positively correlated with transcriptional activity and is considered an epigenetic hallmark of actively transcribed genes [57]. Thus, this specific hypermethylation in the *PCNT* intron might help maintain its transcriptional stability or reflect a higher basal transcription level of this gene under specific cellular states.

*PCNT* encodes a key perinuclear centrosome protein and is critical for normal mitosis and cell proliferation [58]. Liang et al. [30] identified three nonsynonymous mutations in *PCNT* through whole-exome sequencing in patients with Heterotaxy syndrome (HS) complicated by complex CHD, providing preliminary evidence for the role of *PCNT* in the pathogenic mechanism of abnormal heart development. Biallelic loss-of-function (LoF) mutations within the pericentrin (*PCNT*) gene lead to microcephalic osteodysplastic primordial dwarfism type II (MOPD II), which has a phenotypic spectrum that includes ventricular septal defects and acleistocardia [58]. In our study, Gene Ontology (GO) analysis further revealed significant enrichment of DMRs in multiple cellular component processes (cytoplasm, cytosol, membrane and centrosome) involving *PCNT*. Although CHD encompasses diverse phenotypes, structural abnormalities of the heart or great blood vessels are universally associated with cell proliferation.

Furthermore, the Sonic Hedgehog (*SHH*) signaling pathway is considered to be involved in the differentiation and migration of early embryonic cardiac progenitor cells, stimulating and initiating the migration and differentiation of these progenitors to form the atrial and ventricular septa of the heart [59]. In *Shh*^−/−^ mouse embryos, various cardiac malformations have been observed, including multiple CHD phenotypes such as VSD, ASD, and outflow tract defects [31]. *SHH* expression levels in the *SHH* signaling pathway exhibit strict tissue- and developmental stage-specificity; both overexpression and inhibition can lead to cardiac developmental defects [60]. In this study, high methylation of the *SHH* promoter was observed in the discovery cohort, and the corresponding downregulation of expression may have led to abnormal events in cardiac formation during early embryonic development in patients with CHD.

Although there is currently no direct research evidence demonstrating the relationship between *PCNT* intron methylation or its elevated expression and the pathogenesis of CHD, research [61] has confirmed that increased levels of pericentrin would cause alterations in centrosome number, structure, and function. This, in turn, would alter mitotic spindle organization and function, leading to chromosome missegregation. Furthermore, studies have confirmed the involvement of *PCNT* in ciliary transport regulation [62]. Since cilia serve as the core site for Sonic Hedgehog signaling, dysfunction of *PCNT* may lead to cardiac developmental anomalies by disrupting cilia-dependent *SHH* signaling.

However, it must be emphasized that the association between methylation and gene expression is highly context-dependent and tissue-specific. In particular, the relationship between intron hypermethylation and transcriptional activity is not invariant. For instance, the functionality of enhancers depends on low methylation levels [57], which are typically located within intron regions. Therefore, it is not simply the presence of a 5mC mark itself that governs its relationship to transcription but rather the interpretation of the mark in a particular genomic and cellular context [57]. Our inference that the high methylation of *PCNT* introns may be related to their increased expression is based on the universal pattern observed in previous study [63], as well as on our predictive analysis of related splicing factor binding motifs [56] in this sequence. However, this conclusion requires cautious interpretation, particularly as our data are derived from amniotic fluid rather than cardiac tissue.

It is also important to note that the DNA extracted from amniotic fluid is derived from exfoliated cells from the fetal skin, urinary and gastrointestinal tracts, and respiratory system [64]. Therefore, the methylation profile obtained from amniotic fluid samples represents a composite signal. In our study, the observed methylation patterns of *PCNT* and *SHH* in amniotic fluid may differ from the actual state of the developing heart, which is an inherent limitation when using amniotic fluid as a surrogate tissue for research. Nevertheless, the high methylation pattern of *PCNT* in amniotic fluid, which was robustly associated with CHD in this study, holds significant biomarker value. It may reveal a systemic epigenetic dysregulation state in fetuses with CHD, which could manifest in multiple tissues and ultimately be enriched and captured in amniotic fluid samples. It has been shown that, among other cells that are obtained with the amniocentesis sample, there is a fraction of cells exhibiting stem cell like properties [65], suggesting the potential value of our findings in exploring the systemic pathogenesis of CHD and developing novel prenatal biomarkers for women with a high-risk pregnancy. In the future, we will utilize heart-amniotic fluid paired samples or directed differentiation-induced pluripotent stem cells (iPSCs) to investigate the functional roles of these methylation modifications across different tissues and their contributions to the pathogenesis of CHD.

A few limitations should be acknowledged when interpreting the results of this study. Despite all samples being collected within the second trimester, there were significant difference in sampling gestational age between groups. Additionally, data on important covariates (e.g., maternal smoking and BMI) were incomplete. While matching on core variables (e.g., maternal age and fetal sex) was performed, the potential for residual confounding thus remains, and the results should be considered accordingly. Although the risks associated with amniocentesis are considered minimal, as it is an invasive procedure, it is not completely without complications. Given the potential risks of infection, fetal compromise, and preterm birth [66–68], the clinical implementation of amniotic fluid DNA methylation testing should carefully weigh risks and benefits based on fetal conditions. This methylation assay is suitable for use following a suspicious second trimester ultrasound and is recommended for amniotic fluid samples that have been collected for clinical karyotyping or chromosomal microarray analysis. Therefore, this method should be applicable as an adjunctive diagnostic tool in high-risk pregnancy scenarios or when utilizing residual samples from other clinical amniotic fluid tests. Different subtypes of CHD were included, but it was difficult to analyses biomarkers of CHD by subtype due to the limited sample size. The experimental design of marker screening will not be able to clearly determine the causality of the relationships between the methylation and pathogenesis of CHD. Finally, the mechanism of candidate DNA methylation changes is still unclear, and their impact on the occurrence and progression of CHD needs to be further explored.

In conclusion, our research revealed that there are differences in the DNA methylation status of amniotic fluid during the second trimester of pregnancy between patients with CHD and normal individuals. We also identified a methylated biomarker panel that can be used for prenatal diagnosis of CHD, which has high precision and specificity in identifying both simple and complex cases.

## Conclusion

Our results illustrate that DNA methylation profiles in amniotic fluid differ significantly between fetuses with CHD and normal fetuses. The methylation of PCNT was suggested to be associated with the pathogenesis of CHD. The four-marker methylation panel identified in this study demonstrated high efficacy in distinguishing fetuses with CHD, highlighting its promise as a new adjunctive diagnostic approach for prenatal CHD in high-risk pregnant women. Although the precise functional roles of PCNT in CHD pathogenesis require further elucidation, these findings establish their potential significance as diagnostic biomarkers for CHD.

## Supplementary Information

Below is the link to the electronic supplementary material.

Supplementary Material 1

Supplementary Material 2

Supplementary Material 3

Supplementary Material 4

Supplementary Material 5

Supplementary Material 6

Supplementary Material 7

Supplementary Material 8

Supplementary Material 9

## Data Availability

All data generated or analysed during this study are included in this published article and its supplementary information files.

## Abbreviations

CHD: Congenital heart disease
DMRs: Differentially methylated regions
WGBS: Whole-genome bisulfite sequencing
ROC: Receiver operating characteristic
AUC: Area under the curve
SAS: Second-trimester anomaly scan
TBS: Target bisulfite sequencing
SCHD: Simple congenital heart disease
CCHD: Complex congenital heart disease
GO: Gene ontology
KEGG: Kyoto encyclopedia of genes and genomes
ANOVA: One-way analysis of variance
HS: Heterotaxy syndrome
LoF: Loss-of-function
PCNT: Pericentrin
SHH: Sonic hedgehog
GSS: Glutathione synthetase
CI: Confidence interval
